# Bovine tuberculosis and brucellosis prevalence in cattle from selected milk cooperatives in Arsi zone, Oromia region, Ethiopia

**DOI:** 10.1186/1746-6148-9-163

**Published:** 2013-08-13

**Authors:** Rea Tschopp, Birhanu Abera, Sabi Yao Sourou, Emmanuelle Guerne-Bleich, Abraham Aseffa, Alehegne Wubete, Jakob Zinsstag, Douglas Young

**Affiliations:** 1 Armauer Hansen Research Institute (AHRI), P.O. Box 1005, Addis Ababa, Ethiopia; 2 Food and Agriculture Organization, Sub Regional Office for Eastern Africa, P.O. Box 5536, Addis Ababa, Ethiopia; 3 Swiss Tropical and Public Health Institute, Basel 4002, Switzerland; 4 Asella Regional Veterinary Laboratory, Asella, Ethiopia; 5 Public Health Program, Department of Medicine and Pathobiology, Kansas State University, Manhattan, KS 66506, USA; 6 National Animal Health Diagnostic and Investigation Center (NAHDIC), Sebeta, Addis Ababa, Ethiopia; 7 Centre for Molecular Bacteriology and Infection, MRC National Institute for Medical Research, Mill Hill, London NW7 1AA, UK

**Keywords:** Bovine brucellosis, Bovine tuberculosis, Cattle, Milk cooperatives, Disease prevalence, Ethiopia

## Abstract

**Background:**

Bovine tuberculosis (BTB) and bovine brucellosis are two important milk-borne zoonoses that have been shown to be prevalent to various degrees in Ethiopian cattle.

The study was carried out in four Woredas (districts) around Asella town, Arsi Zone between October 2011 and March 2012 and included 318 small-holders in 13 dairy cooperatives that marketed the delivered milk. The aims of the study were i) to assess the prevalence of the two diseases in cattle in a cross-sectional study, ii) to assess potential risk factors of BTB and brucellosis to humans as well as the knowledge-attitude-practice (KAP) among these farmers towards these diseases.

**Results:**

BTB testing using the comparative intradermal skin test (CIDT) was done on 584 milking cows, out of which 417 were serologically tested for brucellosis using the Rose Bengal Plate Test and reactors confirmed with an indirect ELISA test (PrioCHECK®). The individual animal prevalence was 0.3% (95% CI 0.1% to 1.3%) for BTB, 1.7% (95% CI 0.8% to 3.5%) for brucellosis and 8.9% (95% CI 6.8% to 11.5%) for MAC (Mycobacterium avium complex). Of the 13 milk cooperatives, two had at least one positive BTB reactor and five had animals positive for brucellosis.

Cross-breeds accounted for 100% and 71.4% of the BTB and brucellosis reactors respectively. For both diseases, there were prevalence variations depending on Woreda. No animal was concomitant reactor for BTB and brucellosis.

Raw milk was consumed by 55.4% of the respondents. 79.2% of the respondents reported touching the afterbirth with bare hands. The latter was fed to dogs in 83% of the households. One cow among the herds of the 130 interviewees had aborted in the last 12 months. Among the interviewees, 77% stated knowing tuberculosis in general but 42 out of the 130 respondents (32.3%) did not know that BTB was transmitted by livestock. Less than half (47.7%) of the respondents knew about brucellosis.

**Conclusions:**

Low prevalence of both diseases reflected the potential for the area to compete with the growing milk demand. The authors discussed the possible control strategies for the area.

## Background

Bovine brucellosis and tuberculosis are important zoonoses that have been eradicated or controlled in developed countries but remain prevalent in Sub-Saharan nations. Bovine tuberculosis (BTB) is caused by *M. bovis*, a Mycobacterium closely related to the human pathogen *M. tuberculosis*, both belonging to the Mycobacteria Tuberculosis Complex (MTC) [1]. Brucellosis is a bacterial infectious disease caused by Brucella spp, with *B. abortus* primarily affecting cattle. The disease belongs to the world’s major zoonoses [2]. Both diseases can be readily transmitted to humans via the consumption of raw dairy products and/or close contact with infected animals or animal tissue such as placental membranes in the case of Brucellosis [3-5]. Besides being a public health threat, both diseases can have serious economic impacts not only for the animal owners, but also for the regional and national economies through decreased animal productivity and market/trade impairments [2,4,6].

Field and abattoir studies in the last decade showed a low endemicity of BTB in rural areas of Ethiopia characterized by small holders owning predominantly Zebus (*B. indicus*) [7-9]. In urban and peri-urban areas however, BTB prevalence is much higher [10-12]. An extensive study recently carried out in 88 dairy farms in and around Addis Ababa showed an overall individual animal BTB prevalence of 32%, with prevalence peaks of 90% [13]. The latter farms are more market orientated and keep high numbers of upgraded cattle such as Holstein Friesian (*B. taurus*) and their crosses that are yielding more milk than zebus.

In Ethiopia, brucellosis follows a similar distribution and prevalence to BTB. Multiple small scale studies have confirmed the widespread distribution and the low prevalence at animal level (1.6–3.2%) of bovine brucellosis in cattle kept under traditional husbandry [14-18]. Brucellosis prevalence at the individual animal-level is also generally higher in intensive dairy farm systems in peri-urban and urban areas, ranging between 18.4% in Addis Ababa, the capital city, in 1985 [19] and 22.0% in Chaffa Sate Farm, Wollo zone. However, urban data is sparse and often outdated [20-22]. In the Asella region, prevalences of 4.4% and 14.1% have been previously described in intensive dairy farms [18,23].

Arsi (Oromia region) is one of the highest milk producing zones in the country, producing an estimated 74,018 metric tons milk per year (Arsi zone LDHMO, 2002 E.C.). However, demand for liquid milk and milk products is rapidly increasing, particularly in urban areas, due to population growth, as well as the expansion of cafés, restaurants, hotels and supermarkets. To help boost milk supplies and market access, the Food and Agriculture Organization of the United Nations (FAO) launched in 2006 with various partnerships the GTFS/ETH/067/ITA project in Arsi, Oromia, aiming at scaling up the milk production and value addition in dairy cooperatives and helping in linking these small-holder producers to formal markets in the region. Currently, pre-conditions such as good infrastructure, access to markets, existence of strong extension systems, and the organization of farmers into dairy cooperatives and dairy union are assets for further dairy development in the area [24]. Members of these dairy cooperatives are all traditional small-holders engaged in both cropping and livestock husbandry. They bring fresh milk daily to the cooperative dairy units, which process the milk into butter before selling it and the skimmed milk to the population [24].

Milk from these cooperatives, free of milk-borne diseases, will have public health benefits. Moreover, disease free milk would also have enhanced economical value through competitive advantage in the milk market.

The main aims of this study were i) to investigate the prevalence of BTB and brucellosis in milking cows owned by members of these dairy cooperatives and ii) to assess knowledge-attitude and practices (KAP) of the farmers regarding these diseases. The concept of narratives was then applied for the community’s awareness of the public health risks of the two diseases [25,26].

## Methods

### Study site

**Figure 1:**
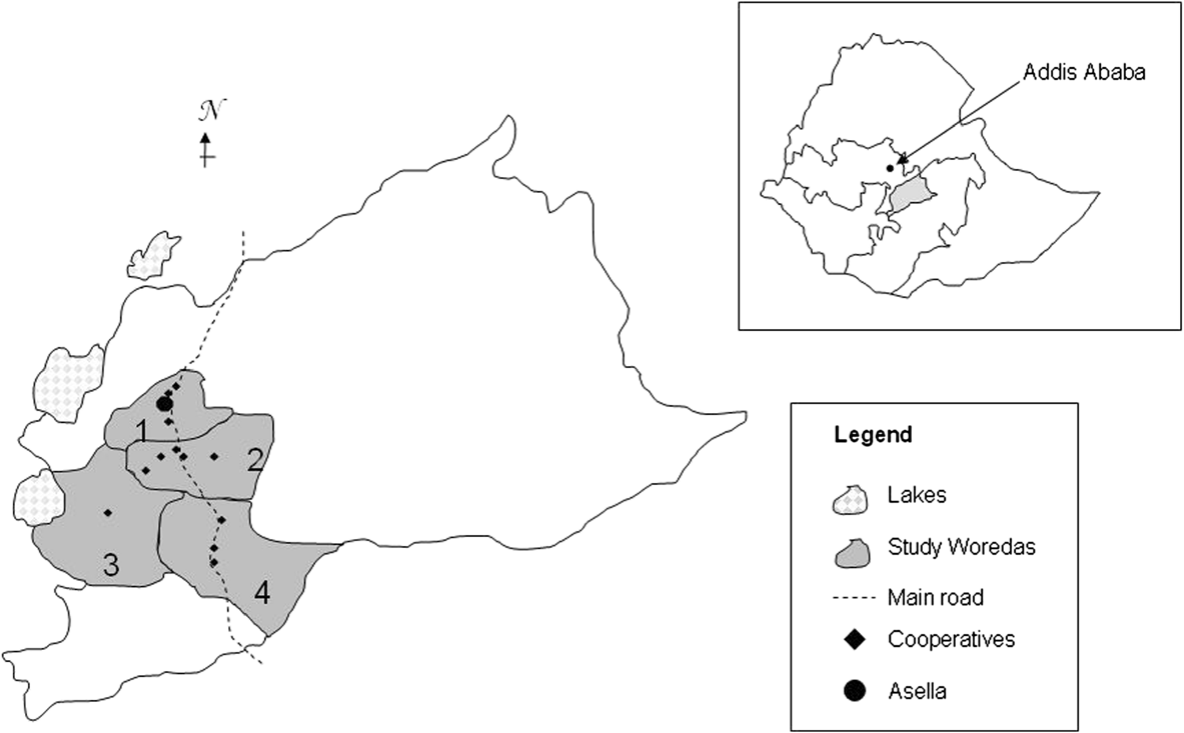
Map of the study area (numbers showing the different Woredas, 1 Tiyo, 2 Digelu Tijo, 3 Munessa, 4 Lemmu Bilbilo) and insert showing in grey shade the Arsi zone within Ethiopia and within the Oromia region in white”.

The study was conducted in Arsi zone, Oromia region, with Asella being the administrative center (Figure 1). The zone is located in South-Eastern Ethiopia at altitude ranging from 1500 to 4245 meters above sea level and is known as the crop belt of Ethiopia thanks to its optimal agro-ecology and flat terrains. It receives biannual rains and the average temperature ranges from 10 to 25°C. The Oromo are the largest ethnic group in Arsi (82.9%) followed by Amhara (15.4%). The majority of the people are Muslim (59.3%), whereas 40% are orthodox Christian. The FAO project area on crop diversification and marketing development covers 8 Woredas out of the 24 in the Zone but functional dairy activity is implemented in only 5 of these Woredas. There are in total 23 dairy cooperatives supplied by 1185 households (FAO communication). The total number of cattle in the project area is 94817. The great majority of farmers are small holders keeping cattle in traditional husbandry systems. Overall zebus are the main cattle breed with crosses making up 14% of the cattle population in the FAO project area (personal communication, H. Ketema, FAO, Arsi). Animals are vaccinated against blackleg, pasteurellosis and anthrax in case of an epidemic outbreak.

### Study design and study animals

We conducted a cross-sectional study between October 2011 and February 2012. Due to considerations of accessibility and distance to laboratory considerations, the study was restricted to the four Woredas (Tiyo, Digelu Tijo, Munessa and Lemmu Bilbilo, Figure 1) involved in the FAO project that were closest to Asella town and represented all four geographical directions (North, South, West and East) around Asella. A stratified cluster sampling proportional to the size of the cattle population was performed in which milk cooperatives were considered clusters. The sampling formula provided by Bennet et al. [27] resulted in a total of 520 animals to be tested for BTB assuming an intraclass correlation coefficient of *rho* 0.2, an expected prevalence of 5% and a standard error of 2.8% [27]. Thirteen dairy cooperatives were randomly chosen out of 21 cooperatives listed by the FAO within the 4 Woredas using random numbers generated in Microsoft Excel®.

Recruitment of animals for testing was based entirely on owner willingness. At each of the participating cooperatives, the cooperative coordinator contacted the cattle owners and asked them to gather their animals at a set time and defined place.

Sampling of animals was restricted to milking cows from owners who were members of the cooperatives and who regularly delivered milk. Excluded due to potential immuno- suppression that would interfere with the skin test were animals showing clinical symptoms of acute diseases, being currently treated for an acute disease or vaccinated for blackleg, pasteurellosis or anthrax in the days prior the skin test. Cows in the last trimester of pregnancy or just having given birth were also excluded for same reason.

After having obtained informed consent from the owners, testing was done, either at the individual household or after gathering animals from all owners in a common place at the milk unit site.

### Bovine tuberculosis and brucellosis testing

Hair on the animal’s neck was shaved at two sites that were 12 cm apart. Following caliper measurements of skin thickness at each shaved site, the site closest to the animal’s head was injected intradermally, using an insulin syringe, with 0.1 ml (2,500 IU/ml) of bovine PPD (Veterinary Laboratories Agency, Weybridge, UK). The other site was similarly injected with 0.1 ml avian PPD. Repeat skin thickness measurements were made 72 hours later. On the reading day, blood was drawn by venipuncture of the jugular vein from animals that could be restrained and whose owners had granted permission. The 10 ml plain vacutainer tubes (Plymouth, UK) into which the blood was collected were centrifuged at the regional veterinary laboratory in Asella; sera were collected in 2 ml cryotubes and frozen until further analysis. Serum was analyzed using the Rose-Bengal Plate Test (RBPT) (Lillidale Diagnostics Badbury View Bothenwood Wimborne Dorset, UK) and positive samples were confirmed with an indirect ELISA for the detection of antibodies against *B. abortus* and *B. mellitensis* in serum and in milk (PrioCHECK® Brucella Ab test, Prionics, Netherlands) at the Armauer Hansen Research Institute (AHRI).

Parameters such as breed, and body condition were recorded for each animal as described in Tschopp et al. [9].

### Questionnaire surveys

A questionnaire survey with open and closed questions was used amongst the owners whose animals were tested and who were willing to participate in the survey. The questionnaire capture information on animal husbandry, milk and meat consumption practices, prevalence of human tuberculosis and brucellosis cases and knowledge, attitude and practice (KAP) regarding both diseases.

### Statistical analysis

Data was double entered in Microsoft Access, compared with EpiInfo software and analyzed with Stata version 10.1 (StataCorp, Texas, USA). Official OIE (Office International des Epizooties) standard for BTB results were used; namely an animal was considered a reactor if the difference between increased skin thickness at the bovine PPD injection site and at the avian PPD injection site was greater than 4 mm. In addition, we used the > 2 mm cut-off for comparison as this cut-off has been suggested to be more appropriate for Ethiopia [28]. MAC (Mycobacterium avium complex) reactors were defined as previously described as animals showing a > 4 mm skin-fold difference at the avium site between day 1 and day 3 (regardless of the bovine reaction) [9]. True prevalence was calculated using both, the Rogan-Gladen estimator and a Bayesian model implemented in the WinBUGS software (http://www.mrc-bsu.cam.ac.uk/bugs/winbugs/contents.shtml). The Bayesian model true prevalence estimation used 30′000 iterations, throwing away the first 10′000 for burn in. The observed number of disease events is assumed to follow a binomial distribution dependent on the true prevalence of disease and the number of observed animals. Test sensitivity, specificity and the true prevalence were specified as Beta probability distributions with prior distribution parameters of 7 and 4 (mode 67) for sensitivity, 10 and 2.5 (mode 86) for specificity and a non-informative prior for the true prevalence.

Animals were considered to be brucellosis positive if they were positive to the RBPT and the ELISA test. Milk cooperatives, treated as a herd, were considered positive if they had at least one positive animal for the specific disease. Univariate analysis was done using logistic regression with disease status as outcome and with random effect on milk cooperatives. Results were shown as OR, 95% for the OR and p-values. Too few BTB reactors with the > 4 mm cut-off meant that the univariate analysis for BTB was done only with the > 2 mm cut-off.

The study has received institutional ethical clearance from the AHRI/ALERT Ethical Review Committee.

## Results

Within the four Woredas, a total of 584 cows from 318 different owners were tested for BTB in 13 milk cooperatives. Minimum and maximum numbers of animals tested per cooperative were 13 and 85 respectively (mean number of animal tested per cooperative: 44.9). Of these, 417 cows in all thirteen cooperatives from 236 owners were tested for brucellosis. The number of animals tested per Woreda is shown in Table 1. 281 (48.1%) were cross breeds (visual inspection coupled with information given by the owner) followed by 266 (45.5%) zebus, and 37 (6.4%) Holstein. While most had a normal body condition score (67.3%), 176 (30.1%) were classified as musculous/fat and 15 (2.6%) as thin.

**Table 1:** Prevalence of BTB (at different cut-offs), of MAC and brucellosis by Woreda

**Diseases**		**Overall**	**Tijo**	**Digelu**	**Munessa**	**Lemmu**
	Total number of cattle*	35798	7762	13756	5955	8325
	Total number of dairy cooperatives**	21	7	6	2	6
BTB & MAC	Number of tested milking cows	584	118	254	43	169
	Number of cooperatives tested	13	4	5	1	3
	Individual BTB prevalence >4 mm (%)	2 (0.3)	0 (0)	0 (0)	1 (2.3)	1 (0.6)
	Individual BTB prevalence > 2 mm (%)	7 (1.2)	1 (0.8)	3 (1.2)	1 (2.3)	2 (1.2)
	Number of BTB positive cooperatives (4 mm)	2 (15.4)	0	0	1	1
	Number of BTB positive cooperatives (2 mm)	7 (53.8)	1	3	1	2
	Individual MAC prevalence (%)	52 (8.9)	5 (4.2)	23 (9)	6 (14)	18 (10.6)
	Number of MAC positive cooperatives	12 (92.3)	3	5	1	3
Brucellosis	Number of tested cows	417	84	197	3	133
	Individual prevalence (%)	7 (1.7)	1 (1.2)	2 (1)	0 (0)	4 (3)
	Number of positive cooperatives	5 (38.5)	1	2	0	2

* Includes all age-sex-breed and purpose (e.g. draft, milk, beef) animals (FAO database).

** Refers to dairy cooperatives within the 4 Woredas and being registered in the FAO project.

### Bovine tuberculosis and brucellosis

Two cross-breed cows out of 584 animals were BTB reactors using the > 4 mm cut-off. Two cooperatives were positive resulting in a cooperative prevalence of 15.4%. The overall individual apparent BTB prevalence was 0.3% (95% CI: 0.1 – 1.3%). Using the > 2 mm cut-off, prevalence increased to 1.2% (95% CI: 0.5 – 3.0%), with 7 reactors and seven cooperatives had reactors, resulting in a cooperative prevalence of 53.8% (Table 1).

The overall individual MAC prevalence was 8.9% (95%CI: 6.8 – 11.5%). BTB prevalence (> 2 mm cut-off) and MAC prevalence were highest in Munessa and lowest in Tijo (Table 1). Assuming published date on sensitivity and specificity for the CIDT, the low apparent BTB prevalence could not be translated into true prevalence using the Rogan-Gladen estimator. Bayesian models showed that the true prevalence was between 0 and 0.5%. Holsteins were significantly more at risk for being BTB reactors compared to zebus (using > 2 mm cut-off) with an OR = 15 (95% OR: 1.3 – 169.4) and p-value: 0.03. Cross-breeds had an increased risk (OR = 3.8; 95% OR: 0.4 – 34.1) but it was not statistically significant (p-value: 0.23). MAC reactors followed a similar trend with Holsteins having an OR = 3.8 (95% OR: 1.4 – 10.0) and p-value: 0.007 and cross breeds having an OR = 2 (95% OR: 1.0 – 3.8) and p-value: 0.04.

Due to the too low number of thin animals, univariate analysis took in account only the categories of “normal” and “musculous” animals. The latter animals showed a higher risk of being reactors than the animals categorized as “normal body score condition” (OR = 1.7; 95%OR: 0.4 – 7.7) but the result was statistically not significant (p-value: 0.5).

The individual bovine brucellosis prevalence in the study area was 1.7% (95% CI: 0.8 – 3.5%). However, there were geographical variations (Table 1) with a maximum prevalence in Lemmu Bilbilo Woreda (3%). Five milk cooperatives had positive brucellosis reactors, giving an overall herd prevalence of 35%. Prevalence variation between the Woredas is shown in Table 1.

No animal was a concomitant reactor to BTB and brucellosis. One animal was brucellosis reactor and MAC positive.

Out of the 7 brucellosis positive animals, 5 were cross-breeds (71.4%) and 2 were zebus. Univariate analysis showed an OR = 2 (95% OR: 0.39; 10.82) for cross-breeds compared to zebus but it was not statistically significant (p-value: 0.38).

### Questionnaire survey

#### Husbandry

A total of 130 owners were interviewed, based on their willingness to participate in the survey. Of these, 97 (74.6%) kept small ruminants with sheep accounting for 61.5%. The majority (94%) kept their animals on communal pastures. Mainly natural service (56%) was used as breeding strategy. Fourteen percent used artificial insemination (AI) and 30% used both AI and natural service for breeding. 12% of households had purchased cattle in the last 12 months.

Over half of the respondents (55.4%) stated that they drank fresh raw milk only. The majority (77.0%) drank raw milk occasionally during the week, whereas 22.3% had milk on a daily basis and 0.7% never drank milk. However, all interviewed owners consumed raw milk products (e.g. butter, cheese) on a regular basis. Most (95.4%) of the milk originated from their own cows, the rest was purchased. Two participants also regularly drank milk from small ruminants. Women were mainly in charge of the milking (84.6%). Men, on the other hand were solely responsible for attending sick animals or animals giving birth (45.4%) followed by women solely (15.4%), and the rest by children or children and adults together.

#### Abortion data

In the 12 months prior to the date on which the questionnaire was administered, one cow (0.8%) and seven (5.4%) small ruminants had aborted. The cow and six of the seven small ruminants aborted in late pregnancy. One calf was stillborn and 17 animals (13%) showed uterine membrane retention post partum. The great majority of respondents (79.2%) stated that they regularly touched the afterbirth with their bare hands. Afterbirth was said to be fed to dogs by 83% of the owners whilst 16.5% buried them (the rest were hung in trees or thrown into long drop toilets). 18.5% recorded chronic cough amongst their cattle. Table 2 shows symptoms respondents had in the last 12 months, with headache, chronic generalized ache and chronic cough as the most frequently experienced symptoms.

**Table 2:** Clinical signs experienced chronically by 130 respondents over the last 12 months

**Clinical signs**	**Number (%) respondents**
Cough (continuous for at least 2 weeks)	20 (15.4)
Haemoptysis	8 (6.1)
Fever episodes	14 (10.8)
Weight loss	7 (5.4)
Night sweat	10 (7.7)
Poor appetite	9 (6.9)
Fatigue/weakness	9 (6.9)
Joint pain	12 (9.2)
Headache	32 (24.6)
General body aching	20 (15.4)
Abortion	4 (3)

#### KAP

Among the respondents, 77% said they knew of the disease tuberculosis in general (not making clear distinction between human and bovine TB) but 32.3% did not know that cattle can transmit BTB to people and 63.8% knew that TB can be cured at the hospital. Less than half of the interviewees (47.7%) had heard about brucellosis and 34.9% of those who knew the disease were unaware that it was curable. In the last five years, five respondents (4%) stated that they were treated in clinics for brucellosis and five others for pulmonary tuberculosis. Six respondents (4.6%) said they had had swollen cervical lymph nodes that they had showed to the doctor. Tables 3 and 4 show how respondent considered brucellosis and BTB to be transmitted to people.

**Table 3:** Transmission of BTB as perceived by the 100 respondents who were aware of BTB

**Route of transmission**	**Number of respondents (%)**
Coughing/sputum	69 (69)
Bad air (e.g. confined environment such as house, public transport, restaurant)	39 (39)
Contaminated milk	23 (23)
Contaminated material (e.g. shoes, clothes, car, furniture)	22 (22)
Consumption of contaminated drinks- unspecific (water, alcohol, milk)	15 (15)
Consumption of contaminated food	14 (14)
Did not know	8 (8)
Smelling rotten things	3 (3)
Soil	3 (3)
Consumption of raw meat	2 (2)
Direct contact with infected animals	1 (1)
Cold weather	1 (1)

**Table 4:** Transmission of Brucellosis as perceived by the 61 respondents who were aware of brucellosis

**Route of transmission**	**Number of respondents (%)**	
Did not know	33 (54)	
No transmission	10 (16.4)	
Carrying heavy loads	7 (11.5)	
Consumption of raw meat	3 (4.9)	
Smelling rotten food	3 (4.9)	
Genetic	2 (3.3)	
Infected bull	1 (1.6)	
Contact with infected placenta	1 (1.6)	
Consumption of infected water	1 (1.6)	
God	1 (1.6)	
Sexual intercourse	1 (1.6)	
Contact with animal blood	1 (1.6)	

Knowledge of BTB was related to literacy grade (chi test; p-value: 0.001): among illiterate owners, 47.4% knew about BTB, whereas 76.8% and 96.0% of the knowledgeable owners went to primary school and secondary school respectively. Knowledge of BTB was also related to the Woredas (chi test; p-value: 0.04), with 44.4% of the owners knowing about BTB in Munessa (minimum) compare to 84% in Digelu Tijo (maximum).

Disease prevalence results were shared with the local authorities in Asella, the dairy cooperatives and the farmers involved. KAP outcome from the above questionnaires were used in the creation of three posters (1.5 m × 1 m each) with colored drawings showing three different scenarios of BTB/brucellosis. This concept of “narratives” that follow two major theoretical frameworks (Health Belief Model, and Social Ecological Model) [25,29] was used to offer an awareness campaign piloted in four milk cooperatives and included in total 153 participants (105 men, 35 women, and 13 teenagers) who were farmers, health workers and local authority workers. Financial constraints meant that the campaign was restricted to four cooperatives that were randomly chosen from the cooperative list.

## Discussion

The amount of milk produced, consumed and marketed has increased amongst milk cooperative members of the FAO project as compared to non-members [24]. This being a likely incentive for farmers to join milk cooperatives, their numbers in the area is likely to increase in the future due to better milk value and market access. This implies that greater rigor would need to be employed in the future to ensure that the liquid milk and milk products sold by these cooperatives is safe for public consumption. Furthermore, good quality milk is in turn a prerequisite for increased market access and price of sold milk, which could for instance be promoted by “good milk certificates” from disease free cooperatives [13].

### BTB and brucellosis

Our results showed a very low individual BTB prevalence in dairy cows from those small-holders delivering daily milk to the milk cooperatives in and around Asella. These results are in line with those reported in small-holder mixed farming system across the country [9,30]. However, the results show a strong contrast to the relatively high BTB prevalence in small-holder cattle in peri-urban Central Ethiopia [31]. This difference was surprising given that this latter area has much similarity with our study site in respect to agro-ecology, husbandry, breeds used, history of exotic breed introduction and supply in the area.

Vordermeier et al. (2012) showed that Holstein and their crosses are more susceptible to BTB than zebus [32]. Our study, in line with previous results [9,31] confirmed that Holstein and their crosses were more at risk than zebu for being BTB reactors. However, despite Holstein and their crosses being as numerous in our study area as in Central Ethiopia area, we found a low overall BTB prevalence. This may be partly explained by certain husbandry practices observed. Although 94% of owners kept their animals on communal grazing land, these animals were predominantly Zebus and low grade crosses. The high productive and expensive cows (i.e. Holstein; high grade crosses) were often kept alone in a secluded shed in the house compound and fed and milked there. This was particularly the case in Tijo and Munessa. The explanation lies in the local cultural tradition/local superstition or belief that bad luck/evil eye would be cast upon these animals if seen by strangers or even neighbors causing them to become dry, sick or dying as a consequence (personal communication, M. Hailu, Tijo). This practice likely reduces the risk of contracting the disease from the animals of other owners and thus limits spread of disease. Both BTB cross-breed reactors from this study had been purchased in the last 5 years from the Addis area and were kept secluded since they were highly productive. Knowing the high BTB prevalence in the Addis area [12,13], it seems possible that both animals had contracted the disease before arriving in the study site.

Our results on apparent individual BTB prevalence contrast with the findings of Dinka and Duressa (2011), who reported high cattle BTB prevalence in both the Tijo (15.4%) and Lemu Woreda (16.1%) using the > 4 mm cut-off [33]. Unlike our study in which we focused on milking cows from small-holder mixed farming systems, Dinka and Duressa examined small scale dairy farms and included in their study all animals regardless of sex and age. Taken together, the results of both studies suggest localized differences in BTB prevalence based on production type. This is consistent with other authors who also found higher BTB prevalence in urban and peri-urban intensive dairy farming system [12,13] as compared to small-holder mixed farming systems. A further contributing factor to the difference in results and low individual prevalence in our study may be that, as mentioned above, we only tested milking cows, thus only a small fraction of the herd. Furthermore, Tschopp et al. (2009) have shown that bulls and oxen have a higher risk of being BTB reactors than females [8].

Brucellosis prevalence at individual animal-level in our study was low overall (1.7%) and in line with previous findings from extensive husbandry systems in Ethiopia in general and in Arsi in particular [18,34]. However, geographical hotspots do seem to occur as seen in Lemmu Bilbilo Woreda (3% prevalence). In the small-holder system in our study, the individual prevalence and herd prevalence of brucellosis was nearly six times and two and a half times higher, respectively, than BTB (> 4 mm cut-off) suggesting it to be a bigger animal and public health problem than BTB. Unlike *M. bovis* which is rarely isolated from milk-although known to be secreted in milk- and is not found in milk that has been stored for a couple of days probably due to the competition with lactobacilli [35], brucella spp are present in 80% of the infected animals in the supra-mammary lymph nodes and the mammary glands and are shed in the milk throughout the life of the animal in large numbers [36]. Large milk volumes are processed by the milk cooperatives and one brucellosis positive cow can thus infect the whole milk contingent putting consumers at risk for disease. Farmers are also at risk through handling the afterbirth with bare hands as seen in our study. In addition the inappropriate disposal of infected fetal membrane allows an extensive environmental contamination and spread of the disease.

Despite 1.7% brucellosis prevalence, only one cow out of 584 had aborted in our study and the results showed a relatively low percentage of fetal membrane retention (13%).

No animal had concomitant infection with Brucellosis and BTB. However, our sample size and disease prevalence were too small to make inferences about the biological or microbiological relationship between the two diseases.

MAC prevalence followed BTB prevalence (using the > 2 mm cut-off) with respect to both Woredas and breeds, suggesting a relationship between MAC and BTB that needs further investigation.

Table 2 shows the symptoms experienced chronically over a period of one year. Several of the perceived morbidity characteristics could be an indication of either tuberculosis or brucellosis. Brucellosis however, is a difficult disease to diagnose clinically due to its varied symptoms [4]. In Ethiopia, the difficulty in diagnosis is compounded by hospitals lacking adequate laboratory diagnostic methods. This is true of the regional hospital of Asella, which does not keep records of brucellosis cases and cannot currently confirm clinical diagnosis with laboratory methods (personal communication, B. Gumi). The authors could therefore not assess the likely prevalence of human brucellosis in the area. It is also unclear whether the five respondents stating they were treated for brucellosis, were diagnosed solely on clinical signs. This study highlights the need for improved diagnostics for human brucellosis in health facilities.

### Control strategies

BTB remains largely uncontrolled in Sub-Saharan countries, with only a few nations conducting test-and-slaughter programs [3,37]. Control of both diseases in the animal reservoir is likely to cause a dramatic decrease in human infection; this is particularly the case with brucellosis [4,38]. Eradication programs, coupled with consequent milk pasteurization have been conducted in developed countries for decades. However, these programs are costly and often logistically challenging in developing countries. Tschopp et al. (2012) showed that although BTB causes substantial financial losses to the Ethiopian economy, it remains not cost-effective to run a nation-wide test-and slaughter program due mainly to the high numbers of cattle in Ethiopia (43 million) and the cost for compensation that has to be covered by the State [39]. Marketed BTB cattle vaccination does not exist at this stage and feasible and cost effective alternatives suited to the Ethiopian context have therefore to be found. Ideally, for logistic and financial reasons, any control program should include simultaneously both milk-borne zoonoses BTB and Brucellosis. An integrated approach making use of inter-sectorial collaborative strategies between the human and the animal health sector with government and non-governmental institutions as well as individual farmers/farmer cooperatives is essential. This would include test and slaughter where feasible (logistically and financially), pre-movement testing program of upgraded animals (with certification), abattoir surveillance, promotion of pasteurization procedures ideally at the dairy cooperatives level, animal segregation on farm level and health communication.

Knowledge of diseases is a crucial step in the development of prevention and control measures [40]. To our knowledge, this was the first time that a Social Ecological Approach was used in BTB and brucellosis awareness campaigns in Ethiopia. Despite six decades of efforts of government and non-government institutions to promote dairy farms in the area, our study highlighted that general knowledge of both milk-born diseases was poor. BTB was in general better known by the farmers as compared to Brucellosis which was mostly unknown; this is probably due to the health education campaigns on human tuberculosis, which gets more social and media attention. Respondents seem not to have properly distinguished BTB from human TB and details of BTB transmission and the involvement of cattle reservoir were poorly known.

Our study generated interest in the use of narratives, not only from farmers, but also from local and national authorities, who showed interest in expanding the awareness program beyond the study area. The method is a cost-effective approach to raise awareness about both diseases. However, further follow-up studies are warranted to investigate the efficacy and impact of such campaigns among dairy cooperatives and farmer communities.

## Conclusions

Although more studies on disease prevalence, in particular in the highlighted study area hotspots but also other cooperatives in the whole region are warranted, this study showed that the milking cows of small-holders being members of dairy cooperatives have a low BTB and Brucellosis prevalence. They have therefore the potential to compete in the dairy market with the higher BTB burden farms in Addis Ababa and in its dairy belt.

Since this favorable disease situation is not the result of informed policy, there is no guarantee that it will continue unchanged. The main current danger is importation of the diseases through purchasing upgraded animals (Holstein and their crosses) in high prevalence areas such as Addis Ababa and its dairy belt, and through the rapid increased use of artificial insemination which aims at increasing the cross-breed population in the area. It is therefore an important period of consolidation for these dairy cooperatives to keep the disease burden low with appropriate measures.

## Competing interests

The authors declare that they have no competing interests.

## Authors’ contributions

RT participated in study design, participated in the laboratory analysis, performed data analysis, and drafted the manuscript. BA participated in the sample collection, laboratory work and literature review. AW participated in the laboratory analysis. SS and EGB participated in the elaboration of the posters for the community awareness program, assisted in the conduction of the awareness campaign, and gave inputs on the milk cooperatives. EGB and AA assisted in the organization of the field work and coordination. AA, DY, EGB assisted in study conception and manuscript revision. JZ assisted in analysis interpretation and gave inputs in the final manuscript. All authors read and approved the final manuscript.
